# Bioinformatics-Based Discovery of Therapeutic Targets in Cadmium-Induced Lung Adenocarcinoma: The Role of Oxyresveratrol

**DOI:** 10.1007/s12011-025-04730-x

**Published:** 2025-07-04

**Authors:** Murat Isıyel, Hamid Ceylan, Yeliz Demir

**Affiliations:** 1 Faculty of Science, Department of Molecular Biology and Genetics, Atatürk University, Erzurum, Turkey; 2 East Anatolian High Technology Research and Application Center (DAYTAM), Atatürk University, Erzurum, 25240 Turkey; 3 Department of Pharmacy Services, Nihat Delibalta Göle Vocational High School, Ardahan University, Ardahan, 75700 Turkey; 4 Faculty of Science, Department of Chemistry, Atatürk University, Erzurum, Turkey

**Keywords:** Bioinformatics, Cadmium, Oxyresveratrol, Lung adenocarcinoma

## Abstract

In this study, we establish a comprehensive bioinformatic and experimental platform for revealing the molecular bases of cadmium (Cd)-induced lung adenocarcinoma (LUAD) and the therapeutic efficacy of the natural polyphenol oxyresveratrol (O-RES) against this disease. A total of 116 differentially expressed genes (DEGs) were obtained, including 30 upregulated and 86 downregulated obtained by integrating five LUAD-related transcriptomic datasets from the GEO database. Results of network analysis identified four core hub genes: upregulated (*Mmp9* and *Col1a1*) and downregulated (*Cdh5* and *Pecam-1*). Using the ToppFUN module, functional enrichment analysis described the identified genes in relevant oncogenic. The in vivo validation with a rat model of Cd-induced lung toxicity further confirmed that the expression levels of these hub genes were markedly changed due to the increased level of Cd, which resembled the human LUAD profile. Strikingly, co-administration of oxyresveratrol (O-RES) further completely abolished the transcriptional aberration of this signaling pathway induced by Cd, indicating that O-RES could attenuate carcinogenic signaling in Cd-induced carcinogenesis. Furthermore, molecular docking analysis revealed that O-RES exhibits strong and specific binding affinities to key LUAD-associated targets, including *Mmp9*, *Col1a1*, *Cdh5*, and *Pecam-1*, supporting its multi-targeted therapeutic potential. These findings not only underscore the potential of *Mmp9*, *Col1a1*, *Cdh5*, and *Pecam-1* as robust biomarkers for Cd-induced LUAD but also highlight O-RES as a promising multi-targeted agent for chemoprevention and therapeutic intervention in environmentally triggered LUAD.

**Supplementary Information:**

The online version contains supplementary material available at 10.1007/s12011-025-04730-x.

## Introduction

Lung adenocarcinoma (LUAD), a predominant histological subtype of non-small cell lung cancer (NSCLC), remains a major contributor to cancer-related mortality worldwide despite advances in early detection and surgical management [1]. While surgical resection offers curative potential in early-stage cases, the variability in disease recurrence and survival outcomes among patients with stage I NSCLC highlights the urgent need for more robust prognostic biomarkers [2]. The prognosis of NSCLC patients remains poor due to the high rate of metastasis and therapy resistance, necessitating the identification of reliable biomarkers for early diagnosis and prognostic stratification [3].

Matrix metalloproteinase-9 (*Mmp9*), a member of the gelatinase subgroup of MMPs, has garnered attention for its capacity to degrade type IV collagen, a key component of the basement membrane, thereby facilitating tumor invasion and metastasis [4]. Emerging evidence indicates that *Mmp9* activity, rather than its mere expression level, may more accurately reflect the aggressive biological behavior of LUAD, as elevated *Mmp9* enzymatic activity has been significantly associated with poorer disease-free survival and increased metastatic potential following resection [5, 6]. Recent studies have further elucidated the molecular mechanisms by which *Mmp9* contributes to NSCLC progression. *Mmp9* expression is often regulated by signaling molecules such as protein kinase C zeta (PKCζ), which promotes tumor cell invasiveness via the MAPK/ERK pathway, consequently enhancing *Mmp9* secretion [7]. These findings underscore the relevance of *Mmp9* not only as a mechanistic driver of NSCLC pathogenesis but also as a promising target for prognostic stratification and therapeutic intervention.

Platelet endothelial cell adhesion molecule-1 (*Pecam-1*, or CD31) is a transmembrane glycoprotein broadly expressed on endothelial cells, platelets, and some leukocyte subsets [8]. In addition to being an endothelial cell marker, *Pecam-1* has also been implied in angiogenesis, and signal transduction events [9]. High density of *Pecam-1* expression was found to correlate with advanced tumor stage, lymph node metastasis, and poor overall survival in patients receiving platinum-based chemotherapy [10]. In contrast, other studies showing that increased *Pecam-1* expression, particularly at the transcriptomic level, could display associations with improved survival suggest context-dependent roles that may be influenced by immune infiltration or stromal composition [11]. Moreover, RNA interference targeting *Pecam-1* shows promising therapeutic potency manifested by the downregulation of vascular endothelial growth factor (VEGF) and inhibition of tumor growth in vivo, further supporting *Pecam-1* potential as a therapeutic target [12]. Together, these findings underscore *Pecam-1* as a pleiotropic biomolecule with relevance to both prognostic determination and therapeutic development in lung cancer biology.

Collagen type I alpha 1 chain (*Col1a1*) is a key extracellular matrix protein responsible for maintaining tissue structure and integrity [13]. Recent bioinformatics and transcriptomic analyses have consistently identified *Col1a1* as a hub gene in NSCLC progression, particularly in LUAD [14]. *Col1a1* overexpression has been strongly associated with poor overall and progression-free survival, especially in smokers, suggesting its role in tumor aggressiveness and immune evasion [15]. Moreover, *Col1a1* appears to be intricately linked to the tumor microenvironment and immune landscape. Its expression positively correlates with infiltration levels of immune cells such as CD4 + T cells, dendritic cells, macrophages, and neutrophils and is significantly co-expressed with immune checkpoint regulators such as CD276 [16]. Additionally, *Col1a1* is responsive to hypoxic conditions within the tumor milieu, where its transcription is significantly upregulated, further implicating it in stress adaptation and treatment resistance mechanisms [17]. Cadherin-5 (*Cdh5*), also known as VE-cadherin, is an endothelial-specific adhesion molecule that regulates vascular permeability and integrity, and its dysregulation has been implicated in tumor progression [18]. Recent studies have highlighted the critical role of *Cdh5* in modulating tumor angiogenesis, cellular migration, and immune responses in various cancer types, including LUAD. For instance, Hung et al. (2016) demonstrated that lung cancer cells harboring epidermal growth factor receptor (EGFR) mutations exhibit significantly elevated *Cdh5* expression, mediated through phosphorylation of EGFR and downstream Akt signaling pathways [19]. Notably, *Cdh5* knockdown in these EGFR-mutated cells led to diminished angiogenic, migratory, and invasive capacities, suggesting that *Cdh5* acts as a downstream effector of EGFR-mediated tumorigenicity.

Studies are still devoid of examples for modular analysis of gene expression data for LUAD that limit knowledge for crucial genes that influence lung adenocarcinomas pathogenesis. Since the early detection and treatment for LUAD are keys to preventing the development of cancer, identifying the ideal diagnostic or therapeutic biomarkers is needed. The network medicine has also implicated rapid development, which reinforced the understanding of disease mechanisms and facilitated breakthrough for drug design. In the present study, we integrated five independent microarray datasets related to LUAD from the GEO database (GSE32867, GSE136043, GSE10072, GSE197346, and GSE43458). A comprehensive differential gene expression analysis was conducted using the GEO2R platform, leading to the identification of 116 differentially expressed genes (DEGs), including 30 upregulated and 86 downregulated genes. To better elucidate the molecular pathogenesis of LUAD, a protein–protein interaction (PPI) network was constructed, and the CytoHubba plugin in Cytoscape was used to identify key hub genes. Based on five topological algorithms, four hub genes *Mmp9*, *Col1a1* (upregulated), and *Cdh5*, *Pecam-1* (downregulated) were identified. To validate our in silico findings, in vivo experiments were conducted using a cadmium (Cd)-induced LUAD rat model, demonstrating significant transcriptional alterations consistent with the human dataset. Notably, co-treatment with the natural polyphenol oxyresveratrol (O-RES) markedly reversed the Cd-induced expression changes in all four hub genes, suggesting its chemopreventive potential. This integrative study provides novel insight into environmentally driven LUAD pathogenesis and proposes *Mmp9*, *Col1a1*, *Cdh5*, and *Pecam-1* as potential biomarkers and therapeutic targets.

## Materials and Methods

### Data Collection

Publicly available independent microarray datasets associated with lung adenocarcinoma (LUAD) are obtained from GEO (https://www.ncbi.nlm.nih.gov/geo/, accessed on April 7, 2025) [20]. Our research has focused on five distinct datasets (filters were applied based on the study’s aim, for example: homo sapiens, expression profiles, and LUAD). Comprehensive features of the expression profiles are shown in Table 1.

**Table 1:** Overwiev of the datasets used in this study

Geo accession	Sample size	Platform	References
GSE32867	58 LUAD tumor samples, 58 adjacent non-tumor tissues	GPL6884 Illumina	[21]
GSE136043	5 LUAD tumor samples, 5 adjacent non-tumor tissues	GPL13497 Agilent	[5]
GSE10072	24 LUAD tumor samples, 15 adjacent non-tumor tissues	GPL96 Affymetrix	[22]
GSE197346	12 LUAD tumor samples, 16 adjacent non-tumor tissues	GPL24676 Illumina	[23]
GSE43458	40 LUAD tumor samples, 40 adjacent non-tumor tissues	GPL6244 Affymetrix	[23]

*GEO* gene expression omnibus, *GPL GEO* platform, *LUAD* lung adenocarcinoma

### Identification of Differentially Expressed Genes (DEGs)

DEGs between LUAD samples and adjacent non-cancerous lung tissue samples were identified using the GEO2R tool (https://www.ncbi.nlm.nih.gov/geo2r, accessed on April 7, 2025). A *p*-value < 0.05 and |log2FC|≥ 1 were defined as thresholds for determining upregulated genes. For downregulated genes, the cut off value was set to |log2FC|≤ 1. Common genes shared in all data sets were determined by the Venn diagram created with the Multiple List Comparator web tool (http://www.molbiotools.com/listcompare.html).

### Protein–Protein Interaction (PPI) Network Analysis

A protein–protein interaction (PPI) network was constructed to examine the interactions among DEGs and to determine crucial hub genes [24, 25]. The PPI network was constructed employing the STRING database (Search Tool for Retrieval of Interacting Genes; https://string-db.org/, accessed on April 7, 2025) [26]. A confidence score of ≥ 0.7 was applied to minimize false-positive interactions. Then, the PPI network was deeply analyzed and visualized by using the Cytoscape (version 3.9.1) software [27]. Significant modules were identified from the PPI network using the Molecular Complex Detection (MCODE) clustering algorithm. To identify the hub genes, five topological analysis algorithms from the CytoHubba plugin of Cytoscape, namely Maximal Clique Centrality (MCC), Maximum Neighborhood Component (MNC), Edge Percolated Component (EPC), Degree, and Closeness, were utilized.

### Gene Ontology and Pathway Enrichment Analysis

Gene ontology (GO) enrichment analysis, which incorporated biological process (BP), molecular function (MF), and cellular component (CC) categories, and Kyoto Encyclopedia of Genes and Genomes (KEGG) pathway analysis of overlapping DEGs were conducted using the ToppFun module of the ToppGene online bioinformatics resource (https://toppgene.cchmc.org/enrichment.jsp, accessed on April 7, 2025) [28].

### Molecular Docking

Three-dimensional crystal structures of *Col1a1* (PDB: 1Q7D), *Mmp9* (PDB: 1L6J), *Cdh5* (PDB: 3PPE), and *Pecam-1* (PDB: 5C14) were downloaded from the Protein Data Bank at RCSB (https://www.rcsb.org). These structures were used as the basis for our molecular docking study. AutoDock Vina and AutoDock Tools were used for this analysis [29]. We used AutoDock Tools to define grid box limits for docking with the Col1a1, Mmp9, Cdh5, and Pecam-1 pockets on the binding region. Later, the protein structure was converted to the pdbqt format (AutoDock-Vina-compliant format) with ligand preparation steps, including the charge, root, aromatic, and hydrogen atoms assignment.

For modeled protein preparation, missing hydrogen atoms were first added followed by elimination of water and any nonspecific metal ions. The O-RES structure was downloaded from PubChem (https://pubchem.ncbi.nlm.nih.gov). All the proteins and esculetin were converted from pdb directly to pdbqts in Autodock Vina. Hydrogens and charges were added to protein by AutoDock in docking studies. Grid box size, 40 × 40 × 40 A°, kept with 0.375 Ao spacing and centered at the billed site of genes. The docking studies were conducted using AutoDock Vina. Visualization and analysis of the docked receptor-ligand complexes obtained were performed by Discovery Studio Visualizer (Dassault Systèms BIOVIA 2021).

### In SilicoComparison and Validations

The Gene Expression Profiling Interactive Analysis (GEPIA; http://gepia.cancer-pku.cn/, accessed on April 7, 2025) platform [30] and the University of Alabama CANcer (UALCAN; https://ualcan.path.uab.edu/, accessed on April 7, 2025) interactive web portal [31] were used to compare mRNA expression variations of hub genes between LUAD samples and the corresponding adjacent (histologically normal, tumor-adjacent, but without noticeable abnormalities) non-tumorous lung samples [32]. The interaction between mRNA expression of hub genes and the clinical stage of lung cancer patients, in addition to the protein levels encoded by hub genes, was also examined by using UALCAN database. Lastly, the Kaplan–Meier plotter platform (https://kmplot.com/analysis/, accessed on April 7, 2025) [33] was used to analyze the prognostic effect of hub genes on the overall survival of LUAD patients.

### Animals and Ethics Statement

Healthy male Sprague–Dawley rats (*Rattus norvegicus*, male, 180 g ± 10 g, *n* = 42) used in this study were purchased from the Atatürk University Medical Experimental Application and Research Center (Erzurum, Turkiye). Rats were randomly divided into six groups as follows: control, dimethyl sulfoxide (DMSO), O-RES 100, Cd(II) chloride, Cd(II) chloride + O-RES-50, and Cd(II) chloride + O-RES-100. The control group rats were treated for 14 consecutive days; only physiological saline will be administered orally. DMSO group rats were treated with %10 DMSO. O-RES 100 group rats were treated with O-RES orally at 100 mg/kg/day for 14 days [34]. Cd(II) chloride group rats were treated with Cd(II) chloride orally at 5 mg/kg/day for 14 days [35, 36]. Cd(II) chloride + O-RES-50 group rats were treated with O-RES orally at 50 mg/kg/day and Cd(II) chloride orally at 5 mg/kg/day for 14 days. Cd(II) chloride + O-RES-100 group rats were treated with O-RES orally at 100 mg/kg/day and Cd(II) chloride orally at 5 mg/kg/day for 14 days. All groups were housed in plastic cages under standard conditions (free access to diet and tap water, 22 °C ± 3 °C air condition, 55% humidity, and 12–12-h lighting). All groups were housed in plastic cages under standard conditions (free access to diet and tap water, 22 °C ± 3 °C air condition, 55% humidity, and 12–12-h lighting). On day 14, all rats were euthanized under ketamine/xylazine (3:1) anesthesia, and lung tissues were removed immediately and kept at − 86 °C after washing cold phosphate-buffered saline. All of the experimental procedures were performed under the guidelines outlined by the National Research Council’s Guide for the Care and Use of Laboratory Animals and were approved under protocol no: 2023/9–148.

### Real-Time PCR Analysis

For relative quantification of hub genes mRNA expression, firstly, total RNA was extracted from rat lung tissues using a commercial total RNA extraction kit (Biorad, Hercules, CA, USA) following the manufacturer’s instruction. Then, the cDNA library was synthesized using the iScript cDNA synthesis kit (Biorad, Hercules, CA, USA) following the manufacturer’s recommendation. To detect hub gene expression pairs, specific primers (Table 2) were designed using the Primer3 (https://bioinfo.ut.ee/primer3-0.4.0/, accessed on 7 April 2025) online tool [37]. For relative quantification, SYBR Green-based qPCR assay was performed using SsoAdvanced™ Universal SYBR® Green Supermix (Biorad, Hercules, CA, USA). *Gapdh* (glyceraldehyde 3-phosphate dehydrogenase) (NM_017008.3) was used as housekeeping control. The comparative ΔΔCt method [38] was used for the relative quantification of gene expression.

**Table 2:** Primer sets used in quantitative PCR (qPCR)

Gene symbol	Accession ID	Sequence
Cdh5	NM_001107407.1	F: 5-AGTTTGCCCTGAAGAACGAG-3
		R: 5-TGATGTTGGCGGTATTGTCG-3
Col1a1	NM_053304.1	F: 5-TCCCAACCCCCAAAAACG-3
		R: 5-TATGACTTCTGCGTCTGGTG-3
Mmp9	NM_031055.2	F: 5-ACCTGAAAACCTCCAACCTC-3
		R: 5-TGCTTCTCTCCCATCATCTG-3
Pecam-1	NM_031591.3	F: 5-TGGAACTGGGGACAAAGAAC-3
		R: 5-TGGCAGCGAAACACTAACAG-3
GAPDH	NM_017008.3	F: 5′-AAACCCATCACCATCTTCCA-3′
		R: 5′-ATACTCAGCACCAGCATCACC-3′

### Statistical Analysis

Statistical comparison of data obtained from measurements made in triplicate (for each animal and sample) was evaluated with one-way ANOVA and Tukey’s post hoc test using Prism (GraphPad Software, San Diego, CA) software. The statistically significant differences are pre sented as follows: ns > 0.05 (not significant); *p < 0.05 (significant); **p < 0.01 (very significant); *** or ****p < 0.001 or 0001 (extremely significant).

## Results

### Determination of DEGs

**Fig. 1:**
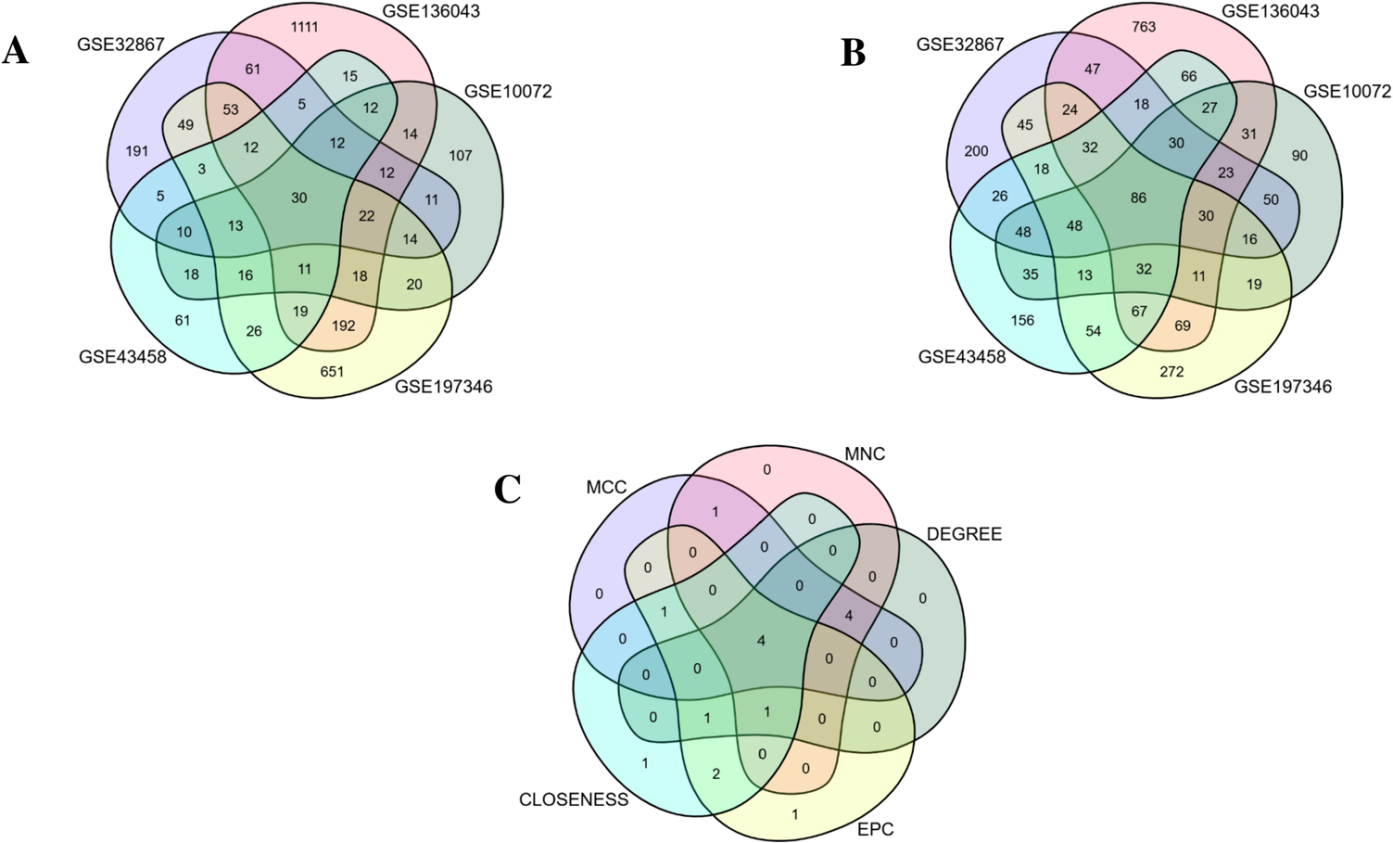
Venn diagrams showing common genes shared between all datasets. **A** Upregulated genes. **B** Downregulated genes. **C** Venn diagram analysis demonstrating the overlapping DEGs among the five algorithms of CytoHubba: MCC, Maximal Clique Centrality; MNC, Maximum Neighborhood Component; EPC, Edge Percolated Component

DEG analysis results showed that there were a total of 116 DEGs shared across all five datasets, comprising 30 upregulated (Fig. 1A) genes and 86 downregulated (Fig. 1B) genes. Detailed information about DEGs is shown in Supplementary Table S1.

### PPI Network and Cluster Analysis

**Fig. 2:**
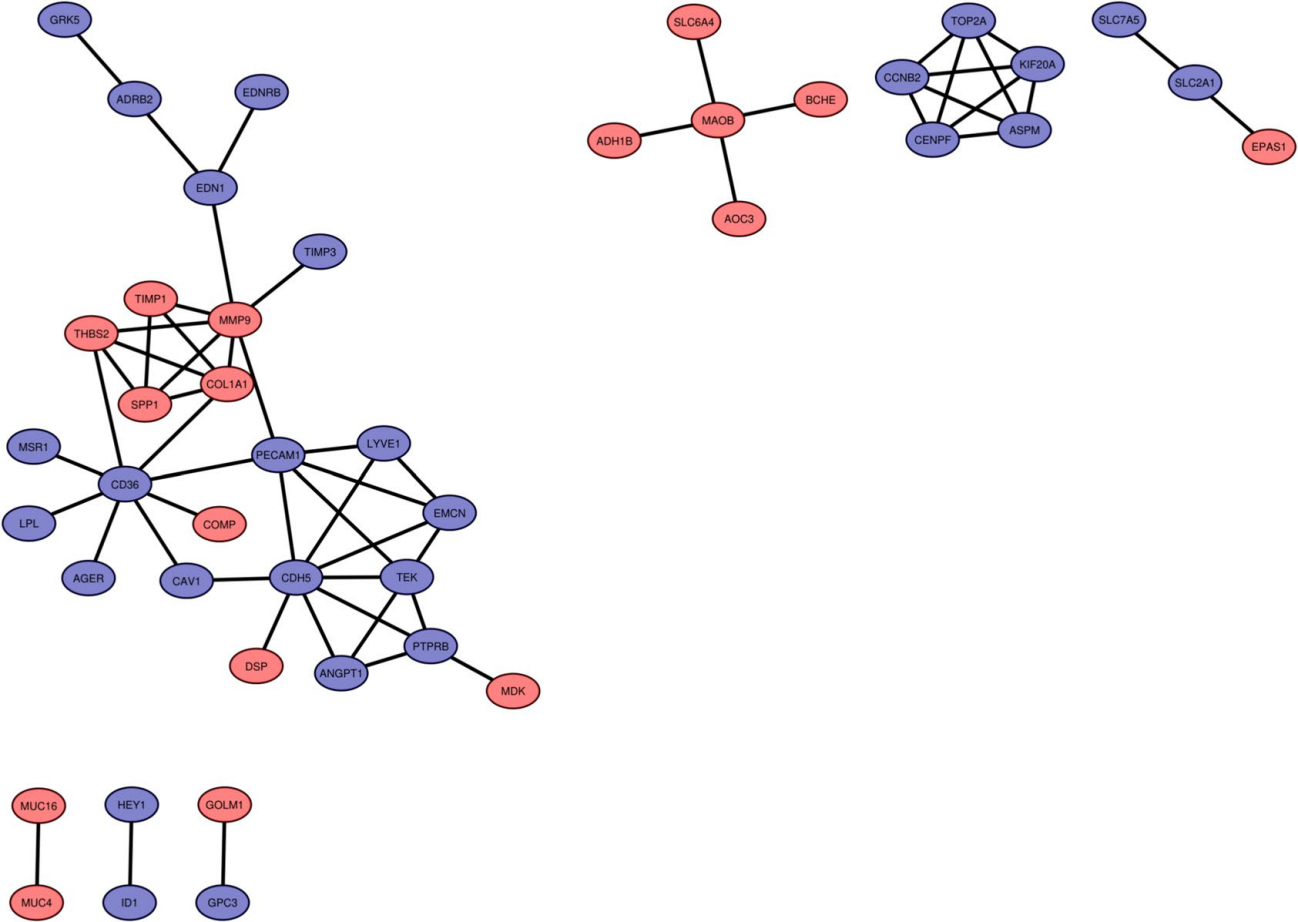
Protein–protein interaction (PPI) network containing all DEGs prominent in LUAD and visualized with Cytoscape. Red nodes represent upregulated DEGs and purple nodes represent downregulated DEGs

**Fig. 3:**
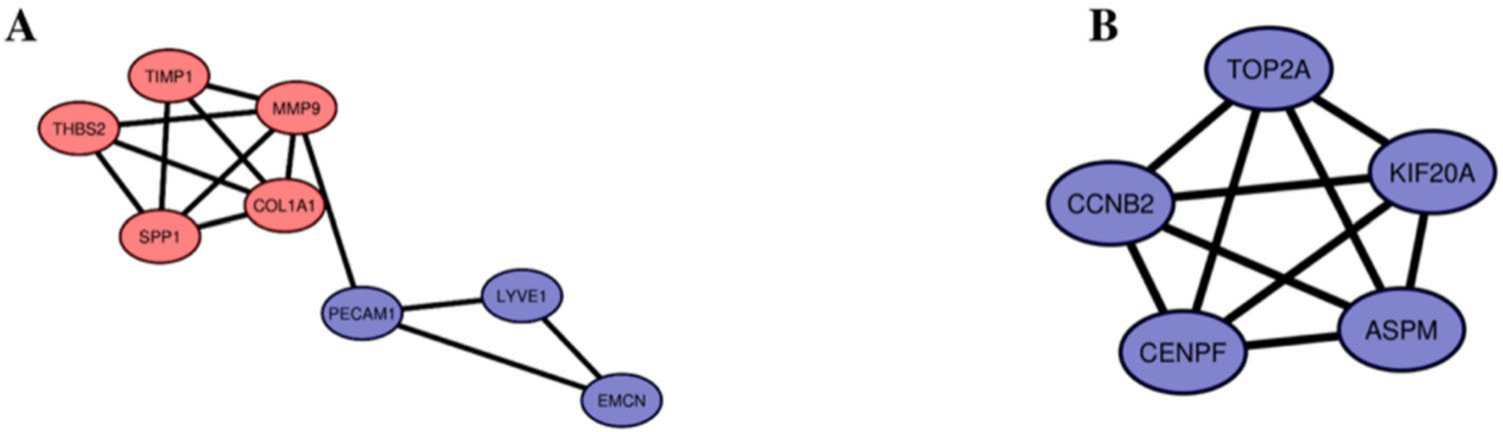
Significant modules identified from the PPI network using the molecular complex detection (MCODE) clustering algorithm. Cluster 1 (**A**) consisting of upregulated and downregulated DEGs and Cluster 2 (**B**) consisting of only upregulated DEGs. Red nodes represent upregulated DEGs and purple nodes represent downregulated DEGs

In the PPI network, which included a total of 44 nodes and 103 interactions (Fig. 2), two clusters were identified (Fig. 3). The top ten genes highlighted by five topological algorithms in CytoHubba were selected. Subsequently, hub gene candidates were determined by identifying shared genes across all algorithms using a Venn diagram (Fig. 1C). Consequently, a total of two downregulated genes (*Cdh5* and *Pecam-1*) and two upregulated genes (*Mmp9* and *Col1a1*) were recognized as hub genes.

### Gene Ontology and Pathway Enrichment Analysis

To explore the roles of 30 upregulated and 86 downregulated DEGs of LUAD, GO and pathway KEGG enrichment analyses were conducted. DEGs were classified into three functional groups of GO: biological processes, cellular components, and molecular functions. Analysis results demonstrated that DEGs identified in LUAD were significantly enriched in molecular functions such as protein and phosphatase binding activities, predominantly involving *Pecam-1* and *Cdh5*. These genes also exhibited strong associations with critical biological processes relevant to cancer progression, including positive regulation of cell migration, motility, and vasculature development. These findings suggest the involvement of these hub genes in essential signaling cascades that facilitate tumor cell invasion, angiogenesis, and metastatic potential, which are hallmark processes in LUAD pathogenesis (Supplementary Table S2).

### Molecular Docking

**Fig. 4:**
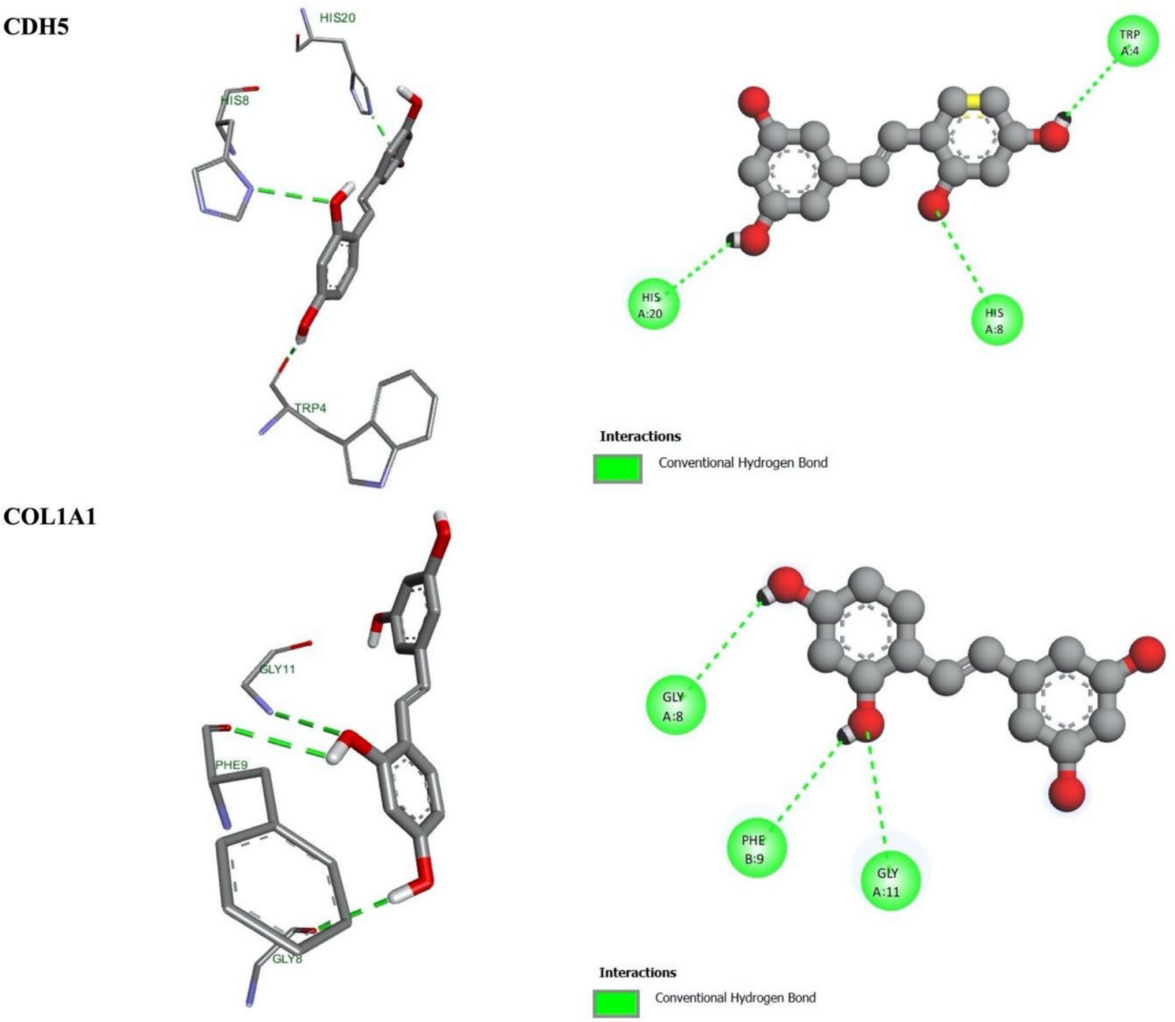
Binding of O-RES with *Cdh5* and *Col1a1* 2D and 3D representation of these proteins showing various interactions

The scoring of O-RES was determined based on its binding mode to the predicted binding site, and the corresponding binding energies are detailed in Table 3. For the genes under study, the molecular docking analysis reveals that O-RES forms multiple conventional hydrogen bonds with key amino acid residues within the active site of the *Cdh5* protein. Specifically, the ligand establishes hydrogen bonds with HIS20, HIS8, and TRP4, suggesting a stable interaction network (Fig. 4). These interactions are predominantly mediated through the hydroxyl and carbonyl functional groups of O-RES, which are oriented towards the polar residues in the binding pocket. The presence of multiple hydrogen bonds indicates a high binding affinity, which may contribute to the inhibitory potential of O-RES on *Cdh5* activity. The interaction with TRP4 is particularly notable, as tryptophan residues often play a critical role in ligand stabilization through π-π or hydrogen bonding interactions. These findings support the hypothesis that O-RES could serve as a promising small-molecule modulator of *Cdh5*, with potential implications in vascular biology and related pathologies.

**Table 3:** Molecular interactions of O-RES with *Col1a1* (PDB: 1Q7D), *Mmp9* (PDB: 1L6J), *Cdh5* (PDB: 3PPE), and *Pecam-1* (PDB: 5C14)

Protein	Ligand	Docking score (kcal/mol)
*Cdh5*	O-RES	− 7.2
*Col1a1*		− 4.6
*Mmp9*		− 9.2
*Pecam-1*		− 6.1

As seen in the docking interaction profiles of O-RES with *Col1a1* protein, three conventional hydrogen bonds of oxyresveratrol with protein residues play an essential role in the stabilization of the ligand in the active pocket. Interestingly, GLY8, GLY11, and PHE9 are connecting to oxyresveratrol to create hydrogen bonds (Fig. 4). Most of these interactions are mediated by the hydroxyl and carbonyl groups of the ligand, inferring a preferred orientation for the *Col1a1* active site polar and non-polar residues. The presence of glycine residues may indicate mobility in the binding site, increasing ligand accommodation. In addition, this unusual hydrogen bonding interaction with PHE9 is likely providing added stabilization through backbone interactions. In brief, such interactions imply that O-RES can promote its modulation of biological activity with *Col1a1* by binding to it in a stable state.

**Fig. 5:**
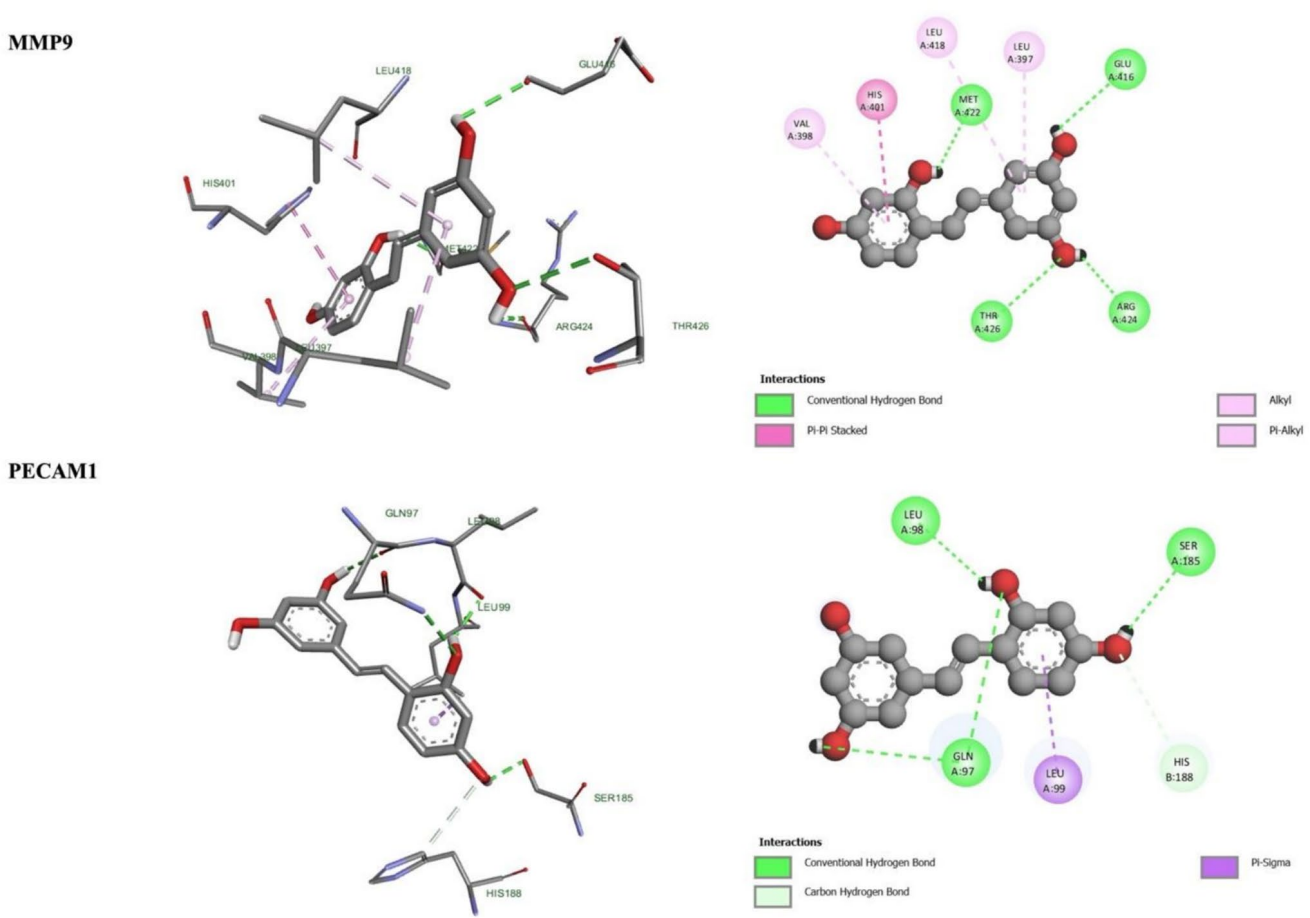
Binding of O-RES with *Mmp9* and *Pecam-1* 2D and 3D representation of these proteins showing various interactions

Docking simulation of O-RES with *Mmp9* displays a multi-contact recognition network through polar and non-polar contacts. Specifically, the classic four hydrogen bonds interactions between the ligand with GLU416, ARG424, THR426, and MET422 are essential for stable binding in the active site of the enzyme (Fig. 5). These interactions are probably mediated via hydroxyl and carbonyl groups of O-RES, confirming it as a hydrogen bond donor/acceptor. Beyond these cases of polar interactions, π-π stacking with HIS401 and hydrophobic interactions that include alkyl and π-alkyl interactions with the side chains of VAL398, LEU397, and LEU418 also provide the ligand with significant, overall stabilization in the *Mmp9* binding pocket. This forms hydrophobic interactions that really anchor the aromatic rings of O-RES enhancing our binding affinity. O-RES forms a good binding orientation with *Mmp9* through hydrogen bonding, π-π stacking, and alkyl interaction, suggesting that it may be a strong inhibitor of *Mmp9*. Overall, this binding profile highlights O-RES as an attractive candidate for potential modulation of *Mmp9*-associated pathophysiological processes, which would be expected to underlie extracellular matrix remodeling and cancer metastasis.

Molecular docking of O-RES with *Pecam-1* shows a stable and complex interaction, mainly consisting of hydrogen bonding and π-interactions. The ligand participates in four typical hydrogen bonds with important polar residues GLN97, LEU98, and SER185 as well as a carbon hydrogen bond with HIS188 (Fig. 5). These polar interactions are crucial for anchoring the ligand inside the active site and aid its stabilization by optimal orientation of hydroxyl and carbonyl moieties. Furthermore, a π-sigma interaction is observed with LEU99, indicating additional non-covalent stabilization facilitated by the aromatic ring system of O-RES. The distribution of hydrogen bonds across different regions of the ligand suggests an extended interaction surface, enhancing the overall binding affinity. These findings collectively suggest that O-RES has the potential to interact favorably with *Pecam-1*, potentially modulating its function in endothelial signaling and inflammation-related pathways.

### In Silico Validations

**Fig. 6:**
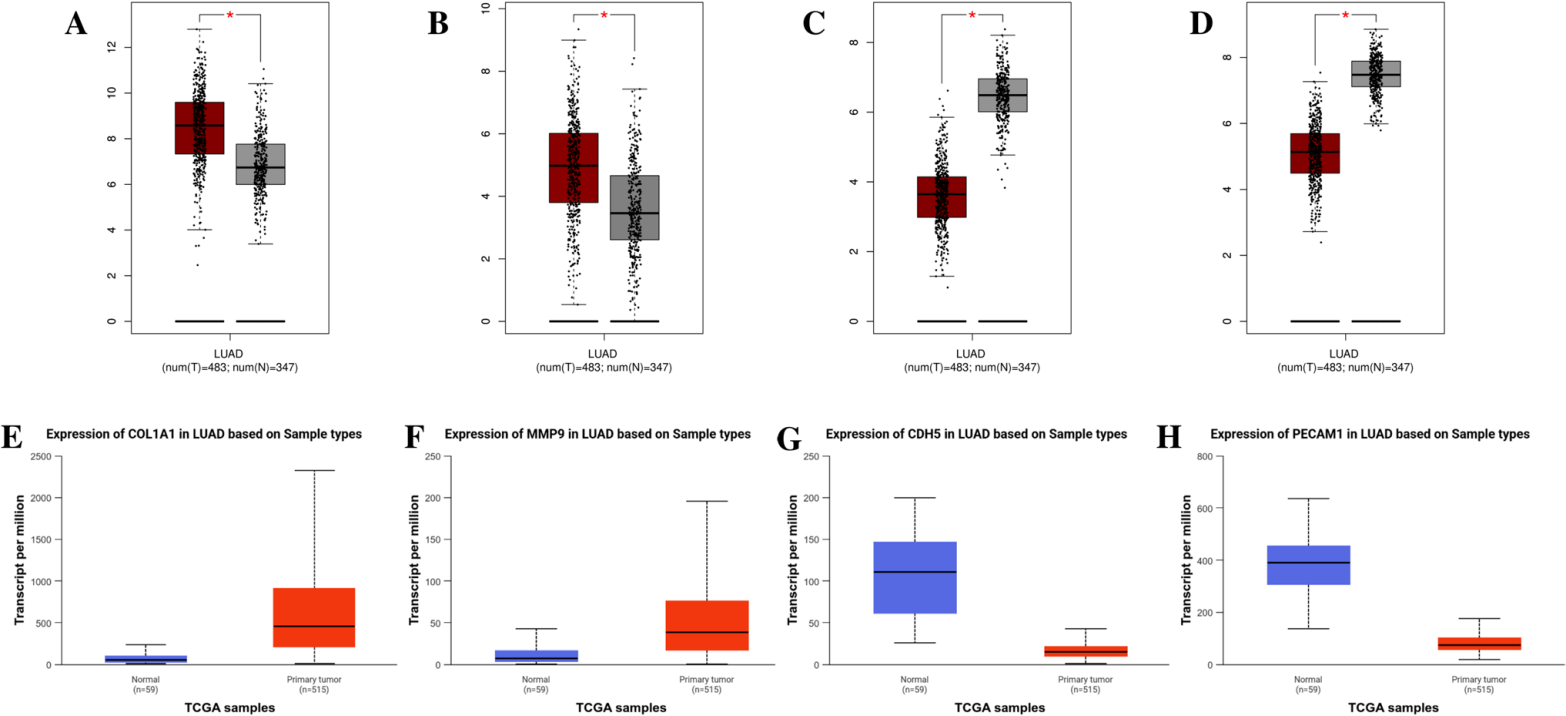
In silico validation results of the hub genes. mRNA expression profiles of hub genes in LUAD according to the GEPIA database: *Col1a1* (**A**), *Mmp9* (**B**), *Cdh5* (**C**), and *Pecam-1* (**D**). mRNA expression profiles of hub genes in LUAD according to the UALCAN database: *Col1a1* (**E**), *Mmp9* (**F**), *Cdh5* (**G**), and *Pecam-1* (**H**)

**Fig. 7:**
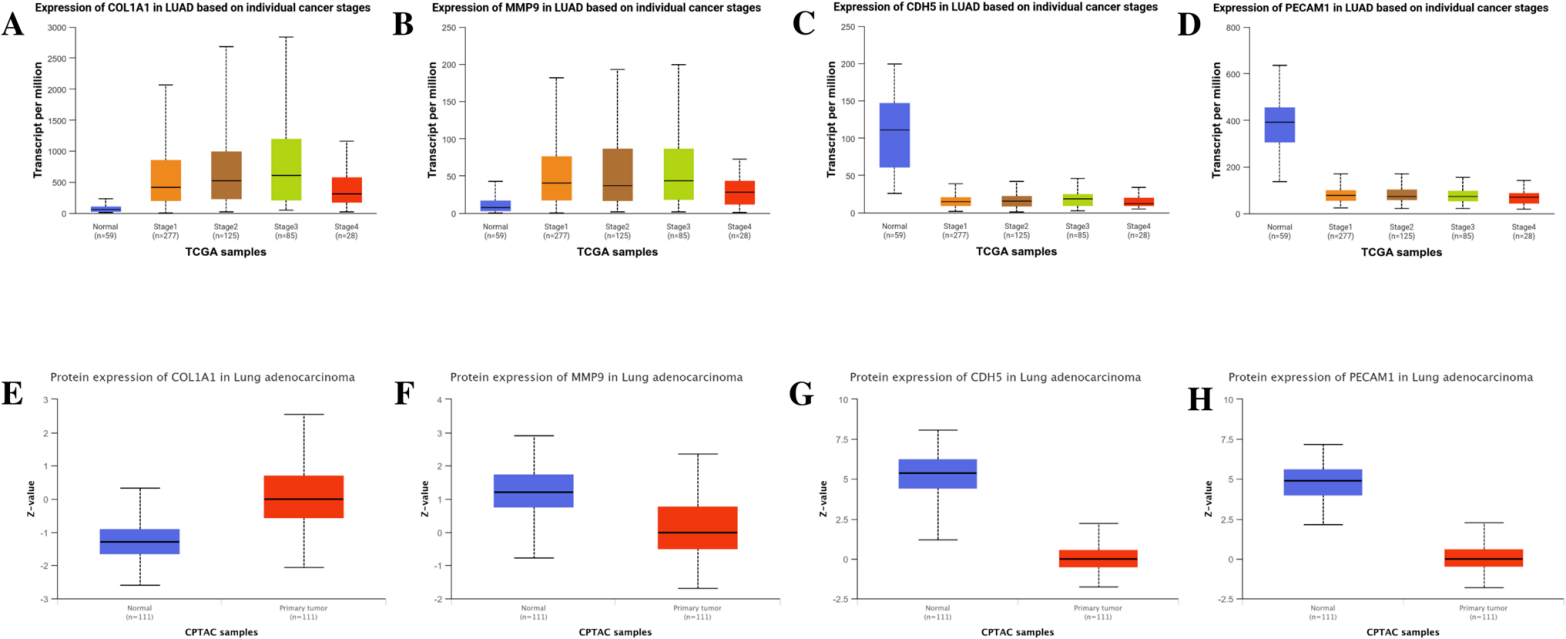
The mRNA expression profiles of LUAD patients in different cancer stages according to the UALCAN database: *Col1a1* (**A**), *Mmp9* (**B**), *Cdh5* (**C**), and *Pecam-1* (**D**). Protein expression profiles of hub genes in LUAD according to the UALCAN database: *Col1a1* (**E**), *Mmp9* (**F**), *Cdh5* (**G**), and *Pecam-1* (**H**)

Consistent with the findings from the GEO dataset analysis, the mRNA expression profiles of *Col1a1* and *Mmp9* genes were found to be upregulated and *Cdh5* and *Pecam-1* in lung tumor tissues against adjacent non-cancerous lung tissues (Fig. 6A–H). Additionally, the expression variations of hub genes at different stages of LUAD were also examined using the UALCAN database. The results demonstrated that the mRNA expression of *Col1a1* and *Mmp9* genes continued to rise and mRNA expression of *Cdh5* and *Pecam-1* continued to decrease in the advanced stages of LUAD (Fig. 7A–D). The mRNA expression profiles of LUAD patients in different cancer stages and protein expression levels of *Col1a1*, *Cdh5*, and *Pecam-1* were found to align with the mRNA expression levels between the lung tissues of LUAD patients and adjacent non-cancerous lung tissues; however, for *Mmp9* protein expression levels, there were differences compared to the other hub genes (Fig. 7E–H).

**Fig. 8:**
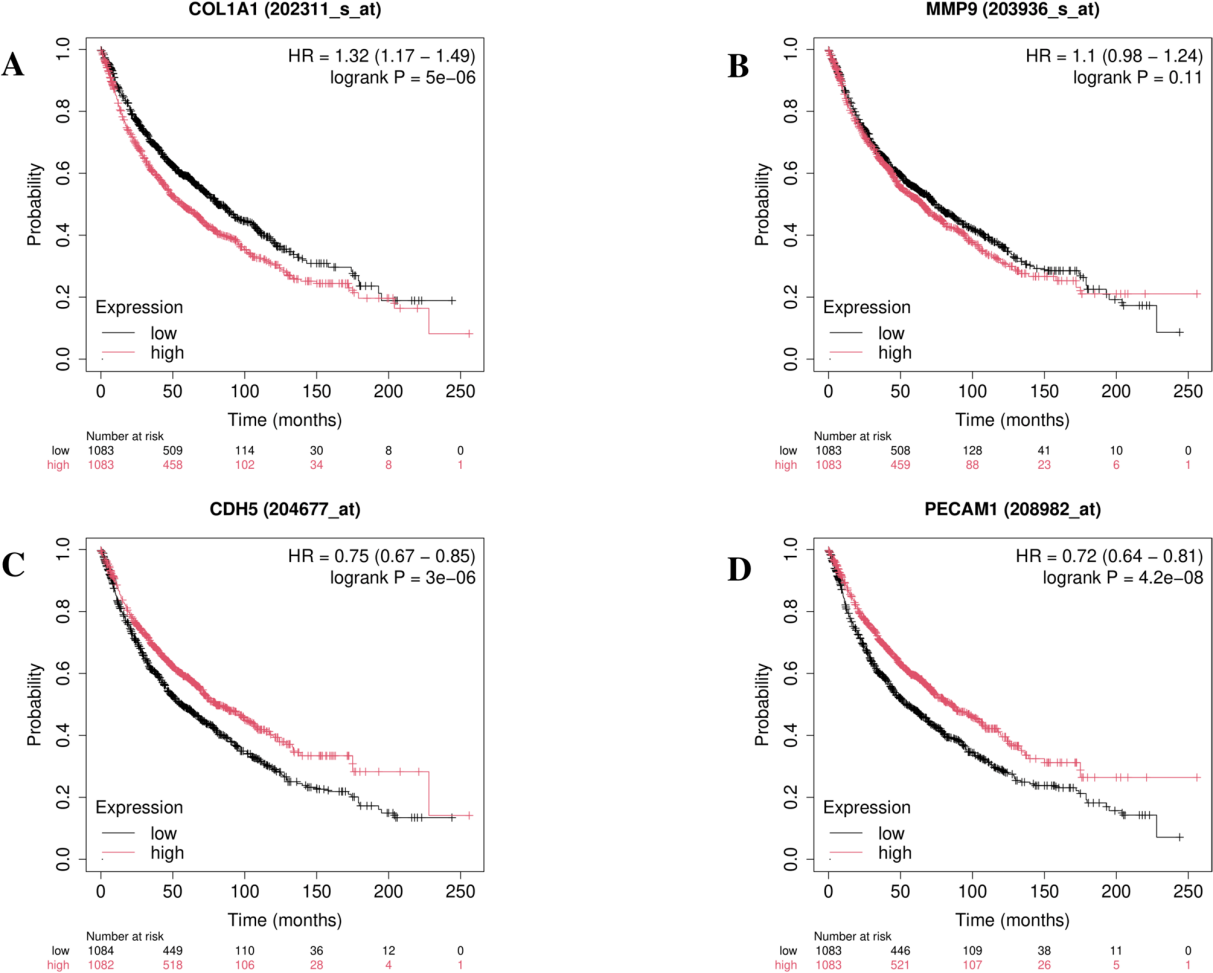
OS analyses of hub genes in patients with LUAD

KM-plotter analysis results showed that increased expression of COL1A and *Mmp9* was linked to poorer OS rates in LUAD patients (Fig. 8A, B). In addition, lower expression of *Cdh5* and *Pecam-1* was also linked to worse OS rates in LUAD patients (Fig. 8C, D). Detailed information on the prognostic values of the identified hub genes for LUAD is provided in Table 4. In conclusion, these results imply that the determined hub genes could function as strong and reliable biomarker candidates for prognostication in LUAD patients.

**Table 4:** Detailed information on the prognostic values of four hub genes in LUAD

Gene	Modulation in LUAD	Affymetrix ID	HR	CI	Log-rank *p*-value	Median survival; low (mo)	Median survival; high (mo)
*Col1a1*	Up	202,311	1.32	1.17–1.49	5e − 06	81	57
*Mmp9*	Up	203,936	1.10	0.98–1.24	0.11	74	65
*Cdh5*	Down	204,677	0.75	0.67–0.85	3e − 06	56	80
*Pecam-1*	Down	208,982	0.72	0.64–0.81	4.2e − 08	54	86

### Gene Expression Analysis by RT-qPCR

**Fig. 9:**
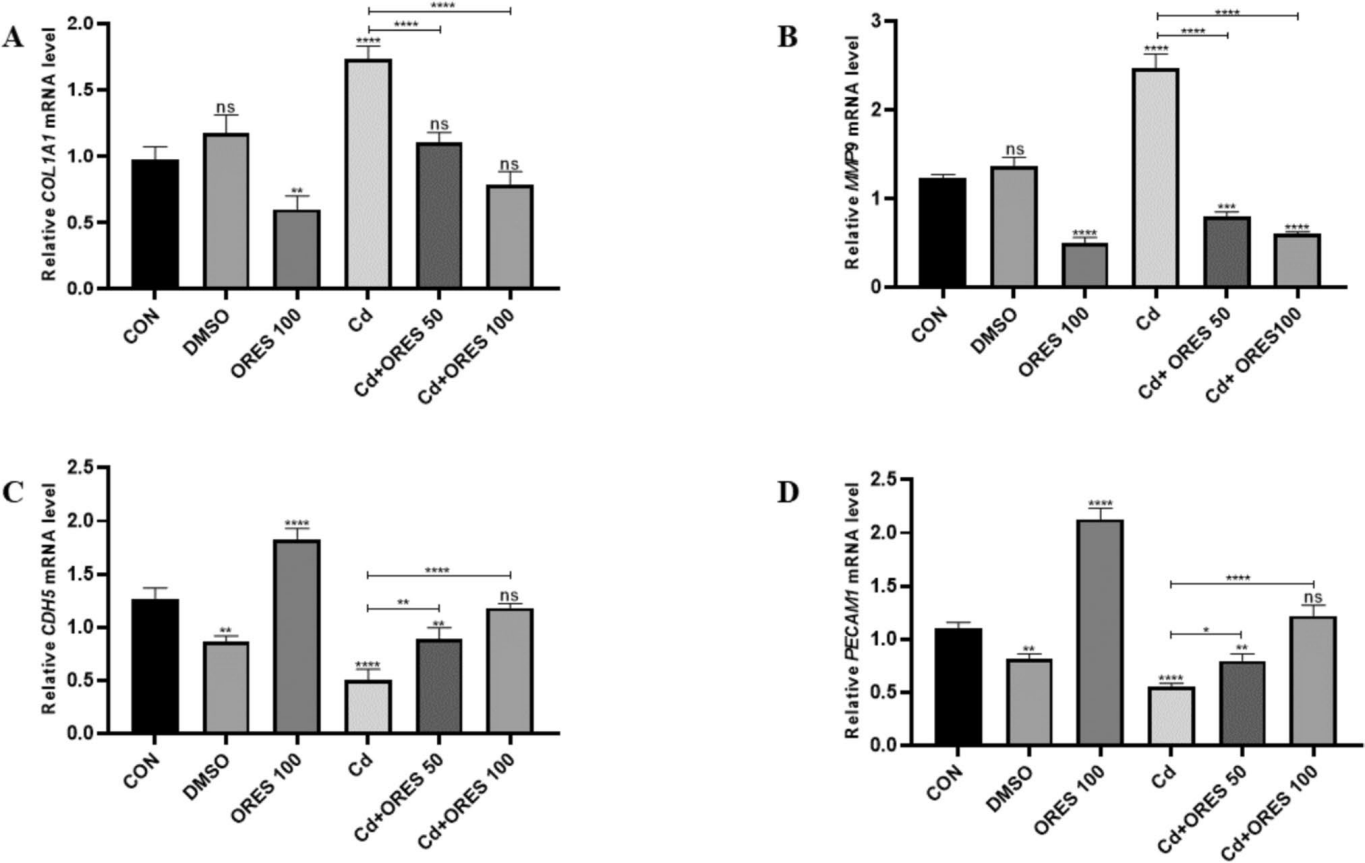
Effects of Cd and O-RES on the mRNA expression levels of key LUAD-related genes in rat lung tissue **A** *Col1a1*, **B** *Mmp9*, **C** *Cdh5*, and **D** *Pecam-1* expression levels were quantified by real-time PCR in lung tissues of rats exposed to Cd alone or co-treated with O-RES (50 or 100 mg/kg/day). CON: untreated control group; DMSO: vehicle control group; ORES 100: rats treated with 100 mg/kg/day O-RES alone; Cd: rats treated with 5 mg/kg/day CdCl_2_; Cd + ORES 50 and Cd + ORES 100: rats co-treated with Cd and 50 or 100 mg/kg/day O-RES, respectively. mRNA levels were normalized to GAPDH expression. Data are presented as mean ± SEM (*n* = 7 per group). Statistical significance was assessed using one-way ANOVA followed by Tukey’s post hoc test. *p* < 0.05 (*), *p* < 0.01 (**), *p* < 0.001 (***), *p* < 0.0001 (****), and ns: not significant

The effects of Cd and O-RES, alone or in combination with each other, on the mRNA transcript levels of hub genes were examined. As shown in Fig. 7A, B, the Cd group (cadmium-treated) shows a significant increase in *Col1a1* mRNA expression compared to the control (CON) and DMSO groups (****p < 0.0001), suggesting Cd may induce a stress response or aberrant chondrogenic-like signaling. Treatment with ORES 100 alone does not significantly alter *Col1a1* levels (ns) compared to control. However, co-treatment with Cd + ORES 50 and Cd + ORES 100 significantly attenuates the Cd-induced increase (****p < 0.0001), suggesting that ORES (presumably a compound with antioxidant or protective properties) mitigates the upregulation of *Col1a1* caused by Cd (Fig. 9A). The *Mmp9* gene, which encodes a matrix metalloproteinase involved in extracellular matrix degradation and inflammation, is strongly upregulated by Cd exposure (****p < 0.0001), indicating tissue remodeling or inflammatory processes are being triggered. Both Cd + ORES 50 and Cd + ORES 100 significantly suppress *Mmp9* expression compared to the Cd group alone, again implying a protective or modulatory role of ORES in Cd-induced responses. ORES 100 alone does not significantly differ from control, further supporting its neutral baseline effect (Fig. 9B).

*Cdh5*, which is essential for vascular endothelial integrity, is markedly upregulated in the Cd group (*p* < 0.0001) compared to control, possibly reflecting a compensatory mechanism or endothelial stress response. Cd + ORES 50 and Cd + ORES 100 significantly reduce *Cdh5* expression relative to the Cd group (*p* < 0.0001 and *p* < 0.01, respectively), suggesting that ORES prevents Cd-induced endothelial alterations. Notably, ORES 100 alone decreases *Cdh5* levels (*p* < 0.01), implying it may modulate endothelial gene expression independently of Cd (Fig. 9C). *Pecam-1*, another endothelial marker implicated in leukocyte transmigration and vascular integrity, is dramatically upregulated by Cd (*p* < 0.0001), likely due to Cd-induced endothelial activation or dysfunction. Cd + ORES co-treatments significantly reduce *Pecam-1* levels compared to Cd alone (*p* < 0.05 and *p* < 0.01, respectively), with Cd + ORES 100 showing a non-significant difference compared to control, highlighting its potential in restoring baseline gene expression (Fig. 9D).

## Discussion

Cd is a poisonous, bioaccumulating, nonessential, and extremely persistent heavy metal associated with numerous detrimental health effects [39]. For non-smoking women not exposed occupationally, food constitutes the primary source of Cd intake, whereas for smokers, inhalation of tobacco smoke is the principal cause of exposure [40]. A minimal proportion of breathed or ingested Cd is eliminated, leading to an accumulation of body burden over time [41]. Epidemiological data from population-based cohorts provide compelling evidence that cadmium exposure is associated with elevated lung cancer risk, with implications for LUAD incidence. A landmark meta-analysis pooling three large prospective cohorts found that each doubling of urinary cadmium was associated with a 68% increase in lung cancer risk [42]. In a Belgian community exposed to environmental Cd near smelters, a doubling of 24‑h urinary Cd was linked to a 70% increased lung cancer hazard, independent of smoking status [43]. Furthermore, meta-analyses that include both general and occupational populations have shown that individuals in the highest cadmium exposure categories exhibit a significantly higher lung cancer risk [44].

In vitro studies present compelling evidence that Cd is linked to human lung cancer (LC) via multiple mechanisms. [45]. LC, the predominant cause of cancer mortality among both men and women globally, accounts for a substantial 18% of all cancer fatalities. Immunotherapy has emerged as an effective treatment for LC [46]. The bulk of adverse reactions were attributable to immunotherapy and can be mitigated with symptomatic treatment, glucocorticoids, and associated monoclonal antibodies [47]. Furthermore, like to other antineoplastic agents, immunotherapy will undoubtedly encounter drug resistance [48]. Consequently, the identification of novel immune-related genes and the evaluation of natural bioactive compounds capable of mitigating cadmium-induced toxicity represent critical priorities in the ongoing effort to understand and counteract LUAD pathogenesis.

Several preclinical studies have investigated the antitumor activity of oxyresveratrol (O‑RES) in lung cancer models, providing important context for its potential effects in Cd‑exposed LUAD tissues. In A549 lung adenocarcinoma cells treated with doxorubicin, O‑RES significantly enhanced cytotoxicity and downregulated genes linked to survival and metastasis, outperforming resveratrol in potentiating chemotherapy efficacy [49]. In a non‑small cell lung carcinoma model, O‑RES induced intrinsic apoptosis and S‑phase cell cycle arrest, evidenced by increased caspase‑3/‑9 activities, mitochondrial membrane potential collapse, and reduced cyclin D expression [50]. More broadly, genome‑wide analyses in A549 and other cancer cells demonstrated that O‑RES modulates key pathways of apoptosis, cell cycle control, and DNA repair, including downregulation of *RAD51* gene, underscoring its multi‑targeted anticancer mechanism [51]. Furthermore, immune-related genes have recently played a crucial role in enhancing prognostics through LUAD transcriptomics microarray, classification, diagnostics, and a comprehensive transcriptome high-throughput sequencing and analysis method that detects alterations in mRNA expression, now employed to elucidate the molecular mechanisms of LUAD [52, 53].

Utilizing whole transcriptome sequencing results from many laboratories enhances statistical power and improves prediction accuracy; additionally, it mitigates the bias inherent in individual research. This work concentrated on the aberrantly expressed mRNAs in LUAD utilizing GEO RNA-seq data, along with the frequent differentially expressed genes identified. There were 30 upregulated DEGs and 86 downregulated DEGs in LUAD with the threshold of ∣log2FC ∣ > 2 and *p* < 0.05. The present study identified and validated four pivotal hub genes *Mmp9*, *Pecam-1*, *Col1a1*, and *Cdh5* in Cd-induced LUAD through integrated bioinformatics and experimental analyses. Each gene contributes to key tumor-promoting processes such as extracellular matrix (ECM) remodeling, immune evasion, angiogenesis, and epithelial-endothelial integrity. Furthermore, their regulation by O-RES provides new insight into phytochemical interventions for LUAD, particularly in the context of environmental carcinogen exposure.

*Col1a1* is recognized for its role in encoding type I collagen, a member of the collagen family that modulates intercellular adhesion and differentiation while fortifying various tissues in the body [54]. Functional enrichment analyses have demonstrated that *Col1a1* is centrally involved in biological processes such as ECM, cell adhesion, and PI3K-Akt signaling key pathways underlying invasion, metastasis, and immune modulation in NSCLC [55, 56]. These findings collectively position *Col1a1* not only as a powerful prognostic biomarker but also as a potential therapeutic target, particularly in the context of immunotherapy and hypoxia-related resistance in LUAD. High *Col1a1* expression has been correlated with poor prognosis, as reported by Pan et al. (2022), who found its overexpression to be significantly associated with reduced overall survival in LUAD [57]. Zhang et al. (2019) showed that the expression of *Col1a1* was significantly elevated in LUAD tissues and that miR‐150/NOTCH3/*Col1a1* axis played a role in EGFR‐TKI resistance in LUAD [58]. Additionally, a previous study showed that *Col1a1* was positively associated with tumor infiltration levels of CD4 + T cells and macrophages in bladder cancer [59]. However, our results revealed an upregulation of *Col1a1* in Cd-exposed lung tissues, which was reversed by the administration of O-RES. Furthermore, the increased levels of *Col1a1* as influenced by Cd indicate a fibrotic-like remodeling of the ECM, reflecting the profibrotic effects of Cd exposure. O-RES exhibits antifibrotic activity as indicated by downregulation of *Col1a1*. O-RES is a naturally occurring stilbene that has attracted considerable attention in the past few years due to its simple chemical structure and many potential pharmaceutical applications [60]. There are studies on the effect of the compounds O-RES derivatives in the treatment of LC. For example, trans-resveratrol derivatives inhibited the expression of JUN and decreased the invasion of LC [61]. In another study, trans-resveratrol was demonstrated to retard A549 cell growth and decrease *Col1a1* expression [62].

*Mmp9* is a type IV collagenase, and high expression of *Mmp9* promotes the progression of LC [63]. Research indicated that *Mmp9* expression was elevated in patients with lung cancer. The serum concentration of *Mmp9* in the experimental group was significantly associated with smoking history, lymph node metastasis, tissue morphology, the TNM stage, and degree of differentiation [64]. Furthermore, Li et al. (2015) indicated that *Mmp9* overexpression correlates with advanced tumor stage and unfavorable prognosis in LUAD [65]. We found consistent upregulation of *Mmp9* across LUAD datasets and Cd-exposed rat lung tissues, which supports previous studies demonstrating that *Mmp9* is a marker of tumor aggressiveness. *Mmp9* was targeted by O-RES, highlighting its potential as a therapeutic target. Increased *Mmp9* expression is directly associated for the metastatic potential and therefore, O-RES treatment mediated downregulation of *Mmp9* may represent normalization of the tumor immune microenvironment, resulting in reduced metastatic potential and enhanced immune surveillance. This is consistent with work by Liu et al. [66] demonstrated that trans-resveratrol can modulate *Mmp9* expression through antioxidative and anti-inflammatory pathways [66].

*Pecam-1* is crucial for vascular homeostasis, transendothelial migration of leukocytes, and immune surveillance. In our study, we analyzed LUAD tissues both in silico and in vivo and found that *Pecam-1* also downregulated significantly in LUAD tissues. This is consistent with the findings of Fei et al. [67], identifying *Pecam-1* as one of the most downregulated hub genes in LUAD. Loss of *Pecam-1* has been associated with increased vascular permeability, abnormal angiogenesis, and enhanced metastasis [68]. As shown by Hung et al. [19], EGFR mutations can promote changes in endothelial gene expression, with *Pecam-1* secretion being a potential downstream effect of this oncogenic pathway. Moreover, in addition to its role in promoting angiogenesis, *Pecam-1* also modulates the interactions between endothelial cells and T lymphocytes [69], and its downregulation may interfere with efficient infiltration of immune cells into the tumor microenvironment. Consistent with its role in promoting endothelial cell function and mobilizing immune cells, we demonstrate that O-RES largely restored *Pecam-1* expression in the endothelium of Cd-exposed rats in our current study. The dual function of *Pecam-1* as a vascular stabilizer as well as an immune regulator also reinforces its potential as a therapeutic biomarker in LUAD [70].

*Cdh5*, an endothelial-specific cadherin, is crucial for maintaining endothelial cell–cell adhesion and vascular permeability. Apart from its structural functions, *Cdh5* has also become an immunomodulatory molecule. Li et al. (2023) found a positive correlation between *Cdh5* and CD8 + T cell activity and immune checkpoint expression, indicating that *Cdh5* plays a role in anti-tumor immunity [71]. Downregulation of *Cdh5* may, therefore, contribute to immune evasion by disrupting T cell-endothelium interactions. In this study, it was significantly downregulated in LUAD tissues and Cd-exposed rat lungs, suggesting a loss of vascular integrity during carcinogenesis. Hung et al. (2016) demonstrated that EGFR mutations lead to upregulation of *Cdh5* in specific NSCLC contexts, enhancing angiogenesis [19]. However, in our Cd-exposed model, the consistent downregulation of *Cdh5* implies a different regulatory mechanism, possibly via oxidative damage to endothelial structures. O-RES restored *Cdh5* expression in our study, suggesting improved vascular integrity and potential normalization of immune interactions. This is consistent with prior studies showing that polyphenols enhance endothelial barrier function through anti-oxidative mechanisms [72]. Hence, *Cdh5* represents both a marker of endothelial dysfunction and a target for immunovascular restoration in LUAD.

Also, in this study, molecular docking analysis provided crucial mechanistic insight into the potential multitargeted therapeutic role of O-RES in Cd-induced LUAD. Among the four core hub proteins identified, *Mmp9* exhibited the most favorable binding energy (− 9.2 kcal/mol), underscoring a strong and stable interaction profile. The docking pose revealed a rich interaction interface consisting of multiple conventional hydrogen bonds (GLU416, ARG424, THR426, MET422) and hydrophobic contacts (VAL398, LEU397, LEU418), as well as π-π stacking with HIS401. These diverse contacts collectively support the hypothesis that O-RES may suppress extracellular matrix remodeling and metastatic signaling via direct inhibition of *Mmp9*. Similarly, *Col1a1* and *Cdh5* were also found to form stable complexes with O-RES via multiple hydrogen bonds with residues such as GLY8, PHE9, HIS8, and TRP4, implying interference with collagen deposition and endothelial adhesion pathways. The interaction between O-RES and *Pecam-1* further suggests potential modulation of vascular integrity and leukocyte transmigration, as the ligand established four hydrogen bonds (GLN97, LEU98, SER185, HIS188) and a π-sigma interaction with LEU99. This intricate binding landscape supports the hypothesis that O-RES may restore immune-vascular dynamics disrupted in LUAD pathogenesis. These findings collectively indicate that O-RES not only binds stably to the four hub proteins but also targets key functional domains involved in cancer progression, thereby reinforcing its candidacy as a multi-modal agent for chemoprevention and therapy in environmentally triggered lung carcinogenesis. Importantly, these in silico interactions corroborate the in vivo transcriptional reversal observed in O-RES-treated LUAD models, suggesting a biologically coherent mechanism of action at both molecular and systemic levels.

The identified expression patterns upregulation of *Mmp9* and *Col1a1* and downregulation of *Pecam-1* and *Cdh5* highlight a convergence of ECM remodeling, angiogenic dysregulation, and immune suppression in LUAD. These molecular events are exacerbated by Cd exposure, reinforcing the environmental contribution to lung carcinogenesis. O-RES was effective in reversing these genetic alterations. Its regulatory effect on all four hub genes indicates a simultaneous modulation of several oncogenic pathways, such as oxidative stress response, PI3K-Akt signaling, and endothelial stabilization. Thus, O-RES might be useful as a multi-targeted chemopreventive agent for especially high heavy metal environmental/occupational exposure individuals.

## Conclusion

The study presents strong evidence for the involvement of *Mmp9*, *Pecam-1*, *Col1a1*, and *Cdh5* in Cd-induced LUAD. In silico bioinformatics tools, independent gene expression databases, and an experimental rat model were employed to validate these genes. Induction of *Mmp9*, *Col1a1* together with repression of *Pecam-1*, *Cdh5* is indicative of a permissive tumor microenvironment, typified by extracellular matrix degradation, immune escape, and vessel hyperpermeability. These genes also demonstrated significant associations with clinical stage and overall survival in LUAD patients, highlighting their prognostic value. Importantly, the administration of O-RES effectively reversed the Cd-induced transcriptional dysregulation of these hub genes, suggesting that O-RES can mitigate the molecular effects of environmental carcinogens. Through antioxidant, anti-inflammatory, and endothelial-stabilizing mechanisms, O-RES appears to counteract key pathways involved in LUAD pathogenesis, including PI3K/Akt, MAPK, and immune suppression pathways. These findings underscore the potential of natural polyphenols as adjunct or chemopreventive agents in lung cancer, particularly in populations exposed to heavy metals. Taken together, this work provides a compelling framework for understanding LUAD pathogenesis in an environmental context while offering novel insights into molecular targets for early detection, prognosis, and intervention. Further mechanistic and clinical studies are warranted to explore the translational applicability of these findings, including the therapeutic viability of O-RES in high-risk cohorts. A limitation of this study is the absence of direct protein-level validation (e.g., Western blot or IHC) for the hub genes due to technical constraints. However, this limitation was partially addressed through in silico proteomic data, molecular docking, and in vivo mRNA analyses. Future studies will aim to expand on these findings through direct protein quantification in lung tissue. Although this study utilized molecular docking to assess ligand–target interactions, future studies may incorporate molecular dynamics simulations to better capture the conformational flexibility and time-dependent stability of O-RES binding to LUAD-related proteins such as MMP9 and COL1A1.

## Supplementary Information

Below is the link to the electronic supplementary material.

ESM 1 (DOCX 31.4 KB)

## Data Availability

Data are available from the corresponding author upon reasonable request.
