# Supplementary material for: Bioinformatics-Based Discovery of Therapeutic Targets in Cadmium-Induced Lung Adenocarcinoma: The Role of Oxyresveratrol
Source: Biol Trace Elem Res. 2025 Jul 4;204(2):1068–83. doi: 10.1007/s12011-025-04730-x (PMC12847123; doi:10.1007/s12011-025-04730-x)
Supplement: Supplementary file 1 — (DOCX 31.4 KB) [file 12011_2025_4730_MOESM1_ESM.docx]

**Supplemantary material**

**Bioinformatics-Based Discovery of Therapeutic Targets in Cadmium-Induced Lung Adenocarcinoma: The Role of Oxyresveratrol**

**Murat Isıyel^1^, Hamid Ceylan^1,2^, Yeliz Demir^3,4*^**

^1^Faculty of Science, Department of Molecular Biology and Genetics, Atatürk University, Erzurum, Türkiye

^2^East Anatolian High Technology Research and Application Center (DAYTAM), Atatürk University, 25240, Erzurum, Türkiye

^3^Department of Pharmacy Services, Nihat Delibalta Göle Vocational High School, Ardahan University, Ardahan, Türkiye

^4^Faculty of Science, Department of Chemistry, Atatürk University, Erzurum, Türkiye

**Table S1.** Common up-regulated and down-regulated DEGs in GEOs derived from LUAD tumor tissues and adjacent non-tumor tissues. **LUAD**; Lung adenocarcinoma, **DEGs**; differentially expressed genes, **GEO**; gene expression omnibus.

| Shared up-regulated (*p*-value <0.05, \|log2FC\| ≥ 1) genes in all GEOs | | | | |
| --- | --- | --- | --- | --- |
| *ASPM* | *COL1A1* | *HMGA1* | *MMP9* | *SLC2A1* |
| *CCNB2* | *COMP* | *HMGB3* | *MUC16* | *SLC7A5* |
| *CDH3* | *CRABP2* | *KIF20A* | *MUC4* | *SMPDL3B* |
| *CEACAM5* | *DSP* | *LGSN* | *PYCR1* | *SPP1* |
| *CENPF* | *GOLM1* | *MDK* | *RGS17* | *SULF1* |
| *THBS2* | *TIMP1* | *TMPRSS4* | *TNFRSF21* | *TOP2A* |
| Shared down-regulated (*p*-value <0.05, \|log2FC\| ≤ 1) genes in all GEOs | | | | |
| *ABCA8* | *CLDN18* | *GNG11* | *MARCO* | *SLIT2* |
| *ACADL* | *CLIC5* | *GPC3* | *MFAP4* | *SLIT3* |
| *ADH1B* | *CTNNAL1* | *GPM6B* | *MME* | *SMAD6* |
| *ADRB2* | *CX3CR1* | *GRK5* | *MSR1* | *SOSTDC1* |
| *AGER* | *DACH1* | *HEG1* | *NDNF* | *SPTBN1* |
| *AHNAK* | *DUOX1* | *HEY1* | *NDRG2* | *STARD13* |
| *ANGPT1* | *EDN1* | *HIGD1B* | *OLFML1* | *STXBP6* |
| *ANXA3* | *EDNRB* | *HOXA5* | *PAPSS2* | *TBX3* |
| *AOC3* | *EMCN* | *HYAL1* | *PECAM1* | *TCF21* |
| *AQP4* | *EPAS1* | *ID1* | *PHACTR2* | *TEK* |
| *BCHE* | *FAM107A* | *JAM2* | *PTPRB* | *TGFBR3* |
| *CA4* | *FERMT2* | *LAMA3* | *S100A8* | *TIMP3* |
| *CALCRL* | *FEZ1* | *LDB2* | *SASH1* | *TMEM100* |
| *CAV1* | *FHL1* | *LIMCH1* | *SEMA5A* | *TNNC1* |
| *CD36* | *FMO2* | *LPL* | *SEMA6A* | *VGLL3* |
| *CDH5* | *FZD4* | *LYVE1* | *SLC39A8* | *VSIG4* |
| *CDO1* | *GHR* | *MAOB* | *SLC6A4* | *WASF3* |
| *WIF1* |  |  |  |  |

**Table S2.** GO (Gene Ontology) and KEGG (Kyoto Encyclopedia of Genes and Genomes) pathway enrichment analysis results of genes found to be differentially expressed (*p*-value <0.05, |log2FC| ≥ 1 and |log2FC| ≤ 1) between LUAD tumor tissues compared with adjacent non-tumor tissues. The top 10 significant GO and pathway terms were listed. **LUAD**; Lung Adenocarcinoma, **MF**; Molecular Function, **BP**; Biological Process, and **CC**; Cellular Component.

| MF: Molecular Function | | | | | |
| --- | --- | --- | --- | --- | --- |
|  | **Term ID** | **Term description** | ***p-*value** | ***q*-value FDR B&H** | **Hit in Query List** |
|  | GO:0019903 | protein phosphatase binding | 0,00065140 | 0,01562000 | PECAM1,CDH5 |
|  | GO:0019902 | phosphatase binding | 0,00102700 | 0,01562000 | PECAM1,CDH5 |
|  | GO:0070051 | fibrinogen binding | 0,00120100 | 0,01562000 | CDH5 |
|  | GO:0043184 | vascular endothelial growth factor receptor 2 binding | 0,00200200 | 0,01691000 | CDH5 |
|  | GO:0048407 | platelet-derived growth factor binding | 0,00240200 | 0,01691000 | COL1A1 |
|  | GO:0070700 | BMP receptor binding | 0,00260200 | 0,01691000 | CDH5 |
|  | GO:0005172 | vascular endothelial growth factor receptor binding | 0,00340100 | 0,01895000 | CDH5 |
|  | GO:0070696 | transmembrane receptor protein serine/threonine kinase binding | 0,00539800 | 0,02632000 | CDH5 |
|  | GO:0033612 | receptor serine/threonine kinase binding | 0,00699300 | 0,02961000 | CDH5 |
|  | GO:0019903 | protein phosphatase binding | 0,00065140 | 0,01562000 | PECAM1,CDH5 |

| BP: Biological Process | | | | | |
| --- | --- | --- | --- | --- | --- |
|  | **Term ID** | **Term description** | ***p-*value** | ***q*-value FDR B&H** | **Hit in Query List** |
|  | GO:0030335 | positive regulation of cell migration | 0,00000125 | 0,00029360 | PECAM1,CDH5,COL1A1,MMP9 |
|  | GO:2000147 | positive regulation of cell motility | 0,00000151 | 0,00029360 | PECAM1,CDH5,COL1A1,MMP9 |
|  | GO:0040017 | positive regulation of locomotion | 0,00000166 | 0,00029360 | PECAM1,CDH5,COL1A1,MMP9 |
|  | GO:0001568 | blood vessel development | 0,00000409 | 0,00051330 | PECAM1,CDH5,COL1A1,MMP9 |
|  | GO:0001944 | vasculature development | 0,00000484 | 0,00051330 | PECAM1,CDH5,COL1A1,MMP9 |
|  | GO:0150105 | protein localization to cell-cell junction | 0,00000777 | 0,00068670 | PECAM1,CDH5 |
|  | GO:0030334 | regulation of cell migration | 0,00001183 | 0,00089550 | PECAM1,CDH5,COL1A1,MMP9 |
|  | GO:2000145 | regulation of cell motility | 0,00001477 | 0,00097830 | PECAM1,CDH5,COL1A1,MMP9 |
|  | GO:0040012 | regulation of locomotion | 0,00001706 | 0,00100500 | PECAM1,CDH5,COL1A1,MMP9 |
|  | GO:0072359 | circulatory system development | 0,00002380 | 0,00116700 | PECAM1,CDH5,COL1A1,MMP9 |

| CC: Cellular Component | | | | | |
| --- | --- | --- | --- | --- | --- |
|  | **Term ID** | **Term description** | ***p-*value** | ***q*-value FDR B&H** | **Hit in Query List** |
|  | GO:0005584 | collagen type I trimer | 0,00038270 | 0,01031000 | COL1A1 |
|  | GO:0030141 | secretory granule | 0,00043870 | 0,01031000 | PECAM1,COL1A1,MMP9 |
|  | GO:0099503 | secretory vesicle | 0,00080750 | 0,01124000 | PECAM1,COL1A1,MMP9 |
|  | GO:0030485 | smooth muscle contractile fiber | 0,00095650 | 0,01124000 | PECAM1 |
|  | GO:0005583 | fibrillar collagen trimer | 0,00229400 | 0,01797000 | COL1A1 |
|  | GO:0098643 | banded collagen fibril | 0,00229400 | 0,01797000 | COL1A1 |
|  | GO:0031092 | platelet alpha granule membrane | 0,00324900 | 0,01886000 | PECAM1 |
|  | GO:0009897 | external side of plasma membrane | 0,00357100 | 0,01886000 | PECAM1,CDH5 |
|  | GO:0062023 | collagen-containing extracellular matrix | 0,00372100 | 0,01886000 | COL1A1,MMP9 |
|  | GO:0098644 | complex of collagen trimers | 0,00401300 | 0,01886000 | COL1A1 |

| KEGG Pathway | | | | | |
| --- | --- | --- | --- | --- | --- |
|  | **Term ID** | **Term description** | ***p-*value** | ***q*-value FDR B&H** | **Hit in Query List** |
|  | M2164 | KEGG_LEUKOCYTE_TRANSENDOTHELIAL_MIGRATION | 0,00000216 | 0,00001943 | PECAM1,CDH5,MMP9 |
|  | M16476 | KEGG_CELL_ADHESION_MOLECULES_CAMS | 0,00052350 | 0,00235600 | PECAM1,CDH5 |
|  | M49004 | KEGG_MEDICUS_PATHOGEN_HBV_HBX_TO_ERK_SIGNALING_PATHWAY | 0,00141800 | 0,00408300 | MMP9 |
|  | M47443 | KEGG_MEDICUS_VARIANT_TMPRSS2_ERG_FUSION_TO_TRANSCRIPTIONAL_ACTIVATION | 0,00198500 | 0,00408300 | MMP9 |
|  | M47780 | KEGG_MEDICUS_REFERENCE_ANGII_AT1R_NOX2_SIGNALING_PATHWAY | 0,00226800 | 0,00408300 | MMP9 |
|  | M19096 | KEGG_BLADDER_CANCER | 0,01187000 | 0,01780000 | MMP9 |
|  | M7098 | KEGG_ECM_RECEPTOR_INTERACTION | 0,02363000 | 0,03038000 | COL1A1 |

| Diseases | | | | | |
| --- | --- | --- | --- | --- | --- |
|  | **Term ID** | **Term description** | ***p-*value** | ***q*-value FDR B&H** | **Hit in Query List** |
|  | C0029172 | Oral Submucous Fibrosis | 0,00000211 | 0,00037710 | COL1A1,MMP9 |
|  | C0005398 | Cholestasis, Extrahepatic | 0,00000683 | 0,00037710 | COL1A1,MMP9 |
|  | C0004364 | Autoimmune Diseases | 0,00001185 | 0,00037710 | PECAM1,COL1A1 |
|  | C0006663 | Calcinosis | 0,00001185 | 0,00037710 | COL1A1,MMP9 |
|  | C0263628 | Tumoral calcinosis | 0,00001185 | 0,00037710 | COL1A1,MMP9 |
|  | C0521174 | Microcalcification | 0,00001185 | 0,00037710 | COL1A1,MMP9 |
|  | C0878659 | Disproportionate short stature | 0,00004019 | 0,00109700 | COL1A1,MMP9 |
|  | C0023893 | Liver Cirrhosis, Experimental | 0,00007051 | 0,00168400 | CDH5,COL1A1,MMP9 |
|  | C0029118 | Opportunistic Infections | 0,00013560 | 0,00235400 | COL1A1 |
|  | C2751322 | Metaphyseal Anadysplasia 2 | 0,00013560 | 0,00235400 | MMP9 |
